# *Lacticaseibacillus rhamnosus* Impedes Growth of *Listeria* spp. in Cottage Cheese through Manganese Limitation

**DOI:** 10.3390/foods10061353

**Published:** 2021-06-11

**Authors:** Lieke A. van Gijtenbeek, Quinn Singer, Louise E. Steffensen, Shannon Neuens, Helle S. Guldager, Susanne Bidstrup, Tina Høgholm, Mikkel G. Madsen, Kathleen Glass, Solvej Siedler

**Affiliations:** 1 Chr. Hansen A/S, Bøge Alle 10-12, DK-2970 Hørsholm, Denmark; dkligi@chr-hansen.com (L.A.v.G.); dklerd@chr-hansen.com (L.E.S.); usshne@chr-hansen.com (S.N.); dkhsg@chr-hansen.com (H.S.G.); dksubi@chr-hansen.com (S.B.); dktho@chr-hansen.com (T.H.); dkmima@chr-hansen.com (M.G.M.); 2 Food Research Institute, University of Wisconsin-Madison, 1550 Linden Drive, Madison, WI 53706, USA; qhuibregtse@wisc.edu (Q.S.); kglass@wisc.edu (K.G.)

**Keywords:** cottage cheese, *Lacticaseibacillus rhamnosus*, *Listeria*, bio-protection, manganese

## Abstract

Acidification and nutrient depletion by dairy starter cultures is often sufficient to prevent outgrowth of pathogens during post-processing of cultured dairy products. In the case of cottage cheese, however, the addition of cream dressing to the curd and subsequent cooling procedures can create environments that may be hospitable for the growth of *Listeria monocytogenes*. We report on a non-bacterio-cinogenic *Lacticaseibacillus rhamnosus* strain that severely limits the growth potential of *L. monocytogenes* in creamed cottage cheese. The main mechanism underlying *Listeria* spp. inhibition was found to be caused by depletion of manganese (Mn), thus through competitive exclusion of a trace element essential for the growth of many microorganisms. Growth of *Streptococcus thermophilus* and *Lactococcus lactis* that constitute the starter culture, on the other hand, were not influenced by reduced Mn levels. Addition of *L. rhamnosus* with Mn-based bioprotective properties during cottage cheese production therefore offers a solution to inhibit undesired bacteria in a bacteriocin-independent fashion.

## 1. Introduction

Consumption of food contaminated with *Listeria monocytogenes* can cause human invasive listeriosis, a disease that poses a high mortality rate for the elderly, pregnant women and their fetuses, newborns, and adults with compromised immunity [1,2]. Although the prevalence of listeriosis outbreaks is very low, severe repercussions make the disease one of the deadliest food-borne illnesses in Europe and the US [1,3]. Bacteria of the genus *Listeria* are known to survive a wide variety of relatively hostile physicochemical environments and, once present, are notoriously difficult to eradicate from food processing plants [4,5,6]. They can form stress-resistant biofilms that are capable of surviving on equipment surfaces consisting of materials such as stainless steel or glass for several years (reviewed in [4]). Vegetative cells can occur in environments with a water activity down to 0.9, salt concentrations up to 12–14%, or at pH values down to 4.3 [7,8,9,10,11,12]. Above all, *L. monocytogenes* is a psychro-trophic organism that not only survives but is also able to maintain growth and even thrive at refrigerated temperatures [12,13,14,15]. The latter poses a specific threat to many ready-to-eat (RTE) foods such as smoked fish products, non-fermented meat, and soft or semi-soft cheeses as refrigeration alone is not enough to protect such foods from listerial growth.

Incidences of listeriosis via cultured dairy products are mostly linked to consumption of soft cheeses prepared from unpasteurized or pasteurized milk, such as brie, camembert, queso fresco, and blue-vein cheeses [16,17,18,19]. For cottage cheese, made with pasteurized milk in particular, the larger threat of *Listeria* contamination lies in the post-fermentation manufacturing steps, when the acidic curd is first heated at 57–62 °C, rinsed with cold water, drained, and subsequently mixed with a chilled cream dressing through which an increase in nutrients and pH as well as an ambient temperature is established. This creates a temporal environment that supports the outgrowth of *Listeria* contaminations that can be introduced during these post-processing steps or carried over from insufficiently pasteurized raw milk [20,21].

Extensive measures are taken to minimize the chances of *Listeria* contamination. These include strict hygienic guidelines during food manufacturing and processing and the addition of preservatives that protect foods during the storage period. While the European Commission (EC) has established a *Listeria* growth potential (δ) criterion that should not exceed 0.5-log colony forming units (CFU)/g in susceptible food products, the United States Food and Drug Administration (FDA), conduits a zero-tolerance policy for *Listeria* counts [22,23]. In this scope, US guidelines state that pasteurized milk products are kept below 7 °C [24]. Because the manufacture of cottage cheese often involves a filling and a cooling step at temperatures up 13 °C, it requires the incorporation of potassium sorbate or another approved food ingredient to prevent growth of *L. monocytogenes* [24]. We will refer to cottage cheese prepared at conditions above 7 °C as “warm filled cottage cheese” throughout the manuscript.

Although synthetic preservatives like sorbate, propionate, benzoate, lactate, and acetate inhibit the proliferation of undesired microorganisms, there is an increasing demand from consumers for more natural solutions and/or clean label products. Especially, food-grade bacterial solutions in the form of live lactic acid bacteria (LAB) have gained popularity. Several LAB have been extensively used in food fermentation for hundreds of years and more recently as probiotics. Many have therefore gained GRAS (Generally Regarded as Safe) status. By taking part in normal fermentation processes, LAB-based food cultures with bioprotective effects help reduce the outgrowth of unwanted contaminants such as *Listeria*, e.g., through the production of weak organic acids, certain secondary metabolites and/or bacteriocins [25,26,27]. 

Weak organic acids such as lactic, acetic, and to a lesser extent propionic acid, are produced by LAB as primary metabolites and acidify the milieu to pH levels not amenable for bacterial growth. However, as *Listeria* spp. display a high resistance to acidic environments [28,29], additional hurdles are required. Secondary metabolites that have been reported to elicit an anti-listerial effect and that can be produced by certain LAB are the amino-acid-derived compounds phenyl-lactic acid, benzoic acid, benzaldehyde, 2-hydroxyl indole-3-propanamide, and indole lactic acid [30,31,32,33,34]. Bacteriocins are small antimicrobial peptides typically produced by bacteria during vegetative growth when nutrient sources are abundant. In this way, bacteriocins help to eliminate other bacteria that are present in the same environment and compete for similar nutrients. Many lactobacilli are known to produce anti-listerial bacteriocins. The so-called pediocin-like class IIa bacteriocins contain the conserved YGNGVXC signature in the N-terminal portion of the peptide sequence and are particularly active against *Listeria* [35]. 

Besides the production of a variety of antimicrobial compounds, bacterial species that inhabit the same niche compete for limiting nutrients. Trace metals such as iron, zinc, copper, and manganese (Mn) are essential for microbial growth and, when present in very low amounts, are a source of competitive exclusion. This pressures bacteria that are specifically sensitive to the depletion of a certain trace element to acquire effective micromineral scavenging systems. For instance, a set of bacteria including many bacilli secrete siderophores for iron scavenging [36]. More recently, elevated expression levels of a Mn-uptake transporter in specific *Lacticaseibacillus paracasei* and *Lacticaseibacillus rhamnosus* strains was shown to be the main responsible mechanism against yeast and mold growth in yoghurts and cheeses [37].

We initiated a screen to identify lactic acid bacteria that help control *Listeria* in warm filled cottage cheese and identified a *L. rhamnosus* strain that protected model cottage cheese from listerial growth. In the current study, we set out to elucidate the mode of action of this strain against *Listeria innocua* and *L. monocytogenes,* and conducted a set of experiments in various model systems to distinguish what type of mechanisms (competitive exclusion, metabolites or bacteriocin production) underpinned the observed anti-listerial activity of *L. rhamnosus* in cottage cheese. We found that the *L. rhamnosus* strain did not produce any anti-listerial bacteriocins when grown in rich medium broth or milk. Rather, it appeared that the growth of *Listeria* was severely hampered by depletion of Mn under growth-permissive pH conditions. This is the first time, to our knowledge, that a potent growth-delaying effect of LAB on *Listeria* spp. is reported based on competitive exclusion rather than bacteriocin production. 

## 2. Materials and Methods

### 2.1. Bacterial Strains, Culture Conditions and Inoculum Preparation

All strains used in this study are summarized in Table 1. The commercial starter cultures FRESCO^®^1000 NG-10 (FNG-10; Chr. Hansen A/S, Hørsholm, Denmark) or FRESCO^®^1000 NG-20 (FNG-20; Chr. Hansen A/S) consisting of *Lactococcus lactis* subsp. *lactis* and *Streptococcus thermophilus* were used to produce cottage cheese. *L. rhamnosus* FreshQ^®^ Cheese +LI 1 (Lrh-FQ; Chr. Hansen A/S) was routinely grown at 37 °C in De Man, Rogosa, and Sharpe broth (MRS). *L. innocua*, *L. lactis* and *S. thermophilus* strains were grown in M17 broth (Difco, BD, Franklin Lakes, NJ, USA) with 0.5% (*w*/*v*) glucose (GM17) alone or in combination with 0.5% (*w*/*v*) lactose (GLM17) as standing cultures at 30 °C. *L. monocytogenes* strains were grown in Brain Heart Infusion (BHI) broth (Oxoid, Thermo Fisher Scientific, Waltham, MA, USA) or Tryptic Soy Broth (TSB; Becton, Dickinson and Company, Sparks, MD, USA) as standing cultures at 37 °C unless stated otherwise. 7.5 µg/mL of chloramphenicol was added to overnight cultures of the red fluorescent *L. innocua* strain to maintain the introduced pEFB001 vector. Stocks of strains were maintained in ceramic beads (CRYO/M; Copan Diagnostics Inc., Murrieta, CA, USA) or via the addition of glycerol, and stored at −80 °C. 

An inoculum consisting of a six-strain mixture of acid-adapted *L. monocytogenes* (FSL R2-501, FSL R2-500, LM101, LM310, LM301 and LM108) was prepared by pre-growing each strain for 24 h in TSB, after which these were diluted 100-fold in fresh TSB supplemented with 1% glucose and incubated until stationary phase was reached to induce acid tolerance [38]. 10 mL of each cell culture was harvested by centrifugation (4000× *g*, 20 min) and suspended in 4.5 mL 0.1% buffered peptone water (BPW; 10 g/L peptone, 5 g/L NaCl, 3.5 g/L Na_2_HPO_4_, 1.5 g/L KH_2_PO_4_, pH 7.1). Strains were mixed in equal concentrations and diluted in BPW to deliver approximately 3.5-log CFU/g to cottage cheese I (see below) using a 0.5% (*v*/*w*) inoculum.

To obtain ampoules with glycerol stocks containing milk-adapted *L. innocua* or a three-strain mixture of milk-adapted *L. monocytogenes* strains (mhl210, ATCC 13932 and DSM 15675), each strain was first grown in PALCAM *Listeria* selective broth (Oxoid, Thermo Fisher Scientific) from a single colony to early stationary phase at 30 °C, diluted 100-fold in standardized boiled milk (B-milk; described in ISO 26323:2009), and grown for another 16 h at 30 °C. The *L. monocytogenes* cultures were mixed in equal volumes. The B-milk cultures were frozen and a CFU count was performed after 24 h of freezing to calculate the cell concentration of the stocks. Prior to inoculation in cottage cheese, 2 mL of a stock ampoule was dissolved in 100 mL B-milk and used to inoculate the various cottage cheeses to establish the indicated CFU counts.

### 2.2. Construction of Red Fluorescent L. innocua

Sequences of oligonucleotides used in this study are shown in Table 2. The broad-range plasmid pEFB001 for constitutive expression of the red fluorescent protein mCherry in Gram-positive bacteria was constructed as follows. The gene sequence for mCherry (GenBank ID AY678264, [39]) was codon-optimized for low-GC LAB using Optimizer [40] with the ‘guided random’ and ‘Codon usage (HEG)’ settings for the *Lacticaseibacillus casei* type strain ATCC334. The P11 promoter is a strong constitutive synthetic promoter developed in *Lacticaseibacillus plantarum* and its sequence was used as originally described [41]. The combined P11 promoter and optimized mCherry gene sequence was ordered as a synthetic construct (GenScript, Piscataway, NJ, USA) and subsequently cloned into the broad host range vector pNZ8148 (MoBiTec GmbH, Goettingen, Germany) as follows. Using Q5 hot start 2× master mix (NEB, Ipswich, MA, USA), P11-mCherry was amplified from the GenScript vector using primers EFB0057+0060 while the pNZ8148 backbone was amplified with primers EFB0061+0062. All primers were purchased from TAG-Copenhagen (Frederiksberg, Denmark) and used at a final concentration of 0.5 µM. The two fragments were gel-purified and joined via Gibson assembly using HiFi 2× master mix (NEB) with incubation for 1 h at 50 °C. The Gibson reaction mixture was then cleaned up using the Monarch PCR & DNA Cleanup kit (NEB) with elution in 15 µL MQ water. 5 µL of this was then used to transform *L. lactis* MG1363 (MoBiTec) via electroporation [42] with a Bio-Rad Gene Pulser (Bio-Rad Laboratories, Richmond, CA, USA), after which cells were plated at 30 °C on GM17 with 5 µg/mL chloramphenicol. Pink/red colonies were obtained and after one transfer of a single colony to a new plate, material was inoculated in 100 mL GM17 with 10 µg/mL chloramphenicol, from which plasmid DNA was extracted using a midi-prep kit (Macherey-Nagel GmbH & Co. Düren, Germany). Pertinent regions were sequenced to confirm their proper nucleotide sequences. Midi-prep material was then used to transform *L. innocua* BL 86/28 using the electroporation protocol routinely used for *L. lactis*. Positive colonies appeared as bright pink/purple colonies, indicating that the mCherry protein is functionally expressed to high amounts. Moreover, these cells were able to grow on *Listeria*-selective PalCam agar plates in the presence of 7.5 µg/mL chloramphenicol.

### 2.3. Agar Well Diffusion Method

*L. rhamnosus* Lrh-FQ was cultivated and diluted 200-fold in fresh MRS or B-milk supplemented with 0.5% (*w*/*v*) tryptone and 0.5% (*w*/*v*) glucose. Cells were grown for 24 h as standing cultures and incubated either aerobically or anaerobically at 37 °C. As a positive control, a sakacin P-producing strain from the Chr. Hansen culture collection was grown to stationary phase in MRS. Inhibitory activity of each culture was then investigated using an agar well diffusion assay. GM17 soft agar (0.75% agar) was inoculated with a 2000-fold dilution of an overnight culture of *L. innocua* grown in GM17 and poured on top of a thin layer of M17 agar in a square dish, leaving out 10-mm holes. 200 µL of each 24-h culture was then added directly into the wells. The plate was incubated at 24 °C for one night after which it was scanned and antimicrobial activity was recorded as growth-free inhibition zones around the wells.

### 2.4. Manufacture of Cottage Cheeses and Inoculation with Listeria spp.

Cottage cheese I. Commercial skimmed milk (~0.05% fat and ~3.2% protein) was pasteurized at 73 °C for 15 s, added to cheese vats and brought to a temperature of 33–36 °C. The tempered milk was then inoculated with 0.26 U/L FNG-10 alone (Vat 1) or in combination with 0.1 U/L *L. rhamnosus* Lrh-FQ (Vat 2). A biological replicate using different lot numbers of FNG-10 and *L. rhamnosus* Lrh-FQ was incorporated (Vat 3). The milk was incubated at 36 °C for ~5 h until a pH of 4.65–4.71 was reached. At this point, the coagulum was cut, healed at 36 °C for 20 min, heated to 43 °C for 1 h and to 57 °C for 30 min with slow continuous stirring (8 rpm). The curd was then drained, washed twice with cold potable water of 13 °C and 2 °C, respectively, under continuous agitation (8 rpm), and drained again before removal from the cheese vat. To prepare the cream dressing, 78% (*w*/*w*) skimmed milk (0.05% fat), 19.8% (*w*/*w*) cream (40% fat) and 2.2% (*w*/*w*) salt (NaCl) were blended, homogenized, pasteurized at 87.5 °C for 25–30 s, and cooled to 3 °C. Creaming was performed by mixing equal parts of curd with cream dressing to create full-fat (4% fat) cottage cheese formulations (~13 °C) in a sterilized stainless steel bin, and pH adjusted to 5.25 ± 0.05 using 10 N HCl. To prevent mold growth that could raise pH and accelerate *L. monocytogenes* growth, 0.002% (*w*/*w*) natamycin powder was added to the rest of the control cottage cheese by dusting over the surface of the finished product and mixing for 3 min before inoculation. The prepared six-strain inoculum of acid-adapted *L. monocytogenes* was mixed for 5 min into each cheese using a sterile stainless steel spatula at a level of 3.5-log CFU/g. The creamed inoculated cheeses were dispensed in triplicate into sterile 60 mL screw-top jars and immediately cooled to and stored at 7.2 °C, representing the FDA National Conference on Interstate Milk Shipments (NCIMS) Pasteurized Milk Ordinance (PMO), or according to Industry Cooling Practices (Exponential cooling from 12.8 °C to 7.2 °C in 72 h, then stored at 7.2 °C).

Cottage cheese II. Commercial skimmed milk eco (Arla; ~0.08% fat and ~3.65% protein) was pasteurized at 70°C for 15 s, cooled to room temperature, added to cheese vats and mixed with 0.0015% (*w*/*v*) CaCl_2_ (CC food^®^ 34%, Tetra Chemicals Europe AB, Helsingborg, Sweden) and 0.0001% (*v*/*v*) of the microbial coagulant Hannilase^®^ XP (Chr. Hansen A/S). The milk was then inoculated with 0.23 U/L FNG-20 alone (Vat 1) or in combination with 0.1 U/L *L. rhamnosus* Lrh-FQ (Vat 2) and incubated at 33 °C for ~5 h until a pH of 4.74 was reached. The coagulum was cut, healed at 33 °C for 30 min, and cooked at 37 °C for 20 min, at 39 °C for 30 min, at 50 °C for 15 min, and at 57 °C for 30 min under continuous stirring for the last three steps. The curd was drained and washed thrice with cold potable water of 12 °C under continuous agitation (60 rpm) and drained again before removal from the cheese vat. To prepare the cream dressing, 80% (*w*/*w*) coffee cream (8.1% fat), 2% (*w*/*w*) cream (38.6% fat) and 2% (*w*/*w*) salt (NaCl) were blended, homogenized, pasteurized at 90 °C for 10 min, and cooled to 3 °C. Creaming was performed by mixing 0.55 parts of curd from Vat 1 or Vat 2 with 0.45 parts of cream dressing to create a cottage cheese formulation (~13 °C) with a pH of around to 5.3 ± 0.5. To half of the cottage cheese, 6 mg/L MnCl_2_ was pipetted on top and mixed in by slowly stirring. The cottage cheeses were distributed as 100-g samples over separate baskets. Three baskets of Vat 1 or Vat 2 were then inoculated with 4-log CFU/g of the milk-adapted 3-strain *L. monocytogenes* mixture and stored according to the warmed filled cottage cheese industry cooling profile (24 h at 12 °C, 25 h at 10 °C and subsequent storage at 7 °C). The remaining portions were inoculated with RFP-tagged *L. innocua* as follows: per gram of cottage cheese, 5 µL of RFP-tagged *L. innocua,* cultivated to stationary phase in GM17 and washed twice with MilliQ to remove residual Mn, was added. Approximately 200 µL of each mixture was dispensed in pentaplicates into a 96-wells to create micro cottage cheeses.

### 2.5. Proximate Analysis of Cottage Cheeses

A proximate analysis was performed on the uninoculated 0-time composite Cottage cheese I and II samples and individual components thereof, including moisture (5 h, 100 °C, vacuum oven method AOAC 950.46 or FoodScan^TM^ Lab, FOSS, Hillerød, Denmark), pH (direct measurement, Orion 8104 combination pH probe and Orion Star A111 pH meter, Thermo Fisher Scientific, Waltham, MA and pH direct measurement, Type 1120, Mettler Toledo, combination pH probe INLAB power PRO-ISM, Mettler Toledo, Columbus, OH, USA), NaCl (measured as % Cl^−^, AgNO_3_ potentiometric titration, Mettler Toledo G20 Compact Titrator), and water activity of Cottage cheese I (AquaLab TE4 water activity meter, Meter Group, Pullman, WA, USA). Results are shown in Table 3.

### 2.6. Manufacture of Cottage Cheese Model (CCM) and Curd with B-Milk Model (CBM)

Commercial skimmed milk (0.1% fat, 3.6% protein) was heat-treated at 90 °C for 5 min, transferred to 200-mL bottles and inoculated with 0.03% (*w*/*w*) FNG-10 starter culture. On top of this, MnCl_2_ and/or *L. rhamnosus* were added to a final concentration of 6 mg/L and 7-log CFU/mL, respectively. The milk was fermented at 35 °C until a pH of 4.6 was reached and heat-treated for 90 min in a water bath at 57 °C. The bottles were centrifuged at 500× *g* for 3 min and the curd and whey (supernatant) were collected. The whey was frozen and the curd was cooled to 12–13 °C and stored at 13 °C before mixing with dressing or B-milk. For the preparation CCM, a cream dressing was prepared by mixing 9% and 38% fat cream to obtain a fat level of about 10.5% and NaCl was added to 2%. The dressing was heat-treated at 90 °C for 5 min, cooled and inoculated with milk-adapted *L. innocua* such that, after mixing 50:50% (*w*/*w*) with a portion of one of the four curds, a final concentration of 1 × 10^3^ CFU/g was established. The samples were stored at 12 °C for 6 days and then at 7 °C for up to 21 days. For the preparation of CBM, a portion of curds obtained from milk fermentations without additional Mn were mixed with B-milk in a 50:50% (*w*/*v*) ratio. The pH was adjusted to a pH of 5.8–6 with 1 N NaOH. The samples were split and 6 mg/L MnCl_2_ was added to one half of the samples. An overnight culture of the RFP-tagged *L. innocua* strain was washed twice with MilliQ to remove residual Mn and used to inoculate one half of the CBM samples by adding 5 µL of this material to each gram of CBM. To the other half, 50 µL/mL of a pH color indicator was added, prepared as described in [43].

### 2.7. Monitoring Bacterial Growth and pH in Cottage Cheese

CFUs of *L. monocytogenes* and *L. innocua* in the various cottage cheeses were determined by diluting and homogenizing a small sample with an equal volume of 0.1% BPW and plating serial dilutions on *Listeria* selective Modified Oxford (Difco, BD) or PALCAM (Oxoid, Thermo Fisher Scientific) agar plates for Cottage Cheese I and II, respectively. The plates were incubated at 30 °C for 2–3 days and *Listeria* was enumerated. The pH was measured on remaining blended, undiluted samples at each sampling time. 

### 2.8. Monitoring Fluorescence and pH in CBM and Micro-Cottage Cheese Models

CBM material with either RFP-tagged *L. innocua* or blue pH indicator dye was divided, in six equal volumes, over two transparent low-well 96-wells plates. The plate containing CBM with RFP-tagged *L. innocua* was incubated at 30 °C for 24 h in a Synergi H1 reader (Bio-Tek, Winooski, VT, USA), during which red fluorescence was followed using mCherry-compatible filters (excitation at 579 nm and emission at 616 nm, as bottom readings with a gain setting of 80). To follow acidification, the plate containing CBM with pH indicator was incubated at 30 °C for 24 h on a flat-bed scanner, after which the color change of the pH indicator dye was converted from hue values to pH as described in [43]. Since the initial start pH values were not all the same, the initial value was set to 0 in order to get a rough comparison of the acidification curves. Red fluorescence of Cottage cheese II samples incubated with RFP-tagged *L. innocua* and incubated at 10 °C were measured using similar settings, but measurements were performed once at indicated days. 

### 2.9. Monitoring Bacterial Growth in Chemically Defined Medium (CDM)

Two types of CDM, one supporting growth of *Listeria* spp. and *L. lactis* (CDM-a) and one supporting growth of *S. thermophilus* (CDM-b), were prepared according to the description in Supplementary Materials Table S1. For both types of CDM, Mn was completely omitted and MilliQ water was used to dissolve ingredients. *L. innocua* BL 86/26, *L. monocytogenes* strains mhl210, ATCC 13932 and DSM 15675, *L. lactis* FNG-10-1 and *S. thermophilus* FNG-10-2 were cultivated to stationary phase in rich nutrient broths after which the cells were thoroughly washed with CDM-a to remove residual Mn. All except *S. thermophilus* FNG-10-2 cells were then diluted 200-fold in CDM-a to which no or a final concentration of 6 × 10^−4^, 6 × 10^−3^, 6 × 10^−2^, 6 × 10^−1^ or 6 mg/L MnCl_2_ was added. For *S. thermophilus* FNG-10-2, the same procedure was carried out in CDM-b. The fresh cultures were divided as 200-µL samples over a 96-well plate, which was covered with transparent sealing tape and incubated at 30 °C in a Synergi H1 reader to follow the growth of each culture. 

### 2.10. Monitoring Growth of L. innocua in Whey

Whey samples collected from milk fermentations for CCM preparation were thawed and mixed 50:50% (*v*/*v*) with MilliQ or CDM-a and 0.5% glucose, adjusted to a pH of 6.5 and filtered-sterilized. To one half of the samples, a final concentration of 6 mg/L MnCl_2_ was added. To monitor growth of *L. innocua* in the various samples, an GM17-based overnight culture was washed twice with MilliQ and diluted 200-fold in each sample. The growth of quadruplicate or pentaplicates samples was followed at 30 °C in a 96-well-plate using a Synergi H1 reader.

## 3. Results

### 3.1. Discovery of Lactic Acid Bacteria Strains with Antilisterial Properties in Cottage Cheese

**Figure 1:**
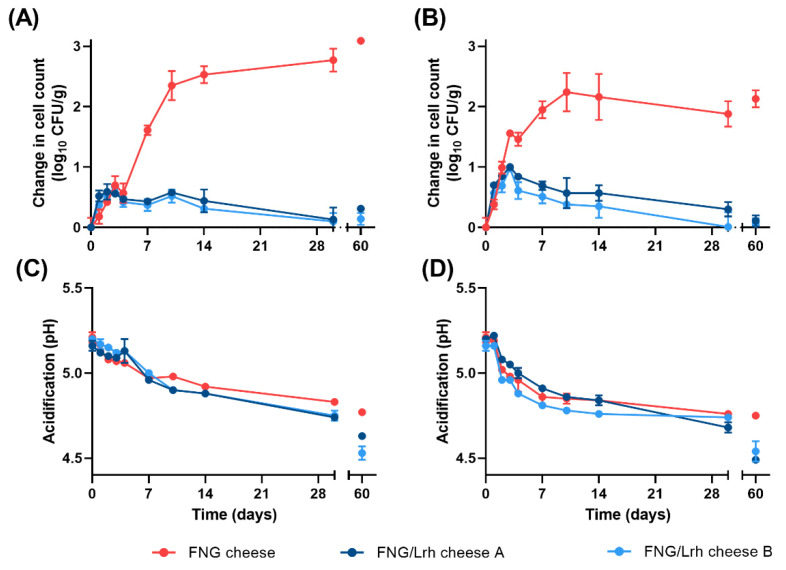
Growth of *L. monocytogenes* and development of pH in cottage cheese prepared with or without Lrh-FQ. Shown are the changes in *L. monocytogenes* CFU/g compared to Day 0 as detected in FNG or FNG/Lrh-FQ cottage cheeses after PMO (**A**) or industrial (**B**) cooling, and corresponding acidification curves of the cheeses after PMO (**C**) or industrial cooling (**D**). Shown are averaged values obtained from measurements in three cheeses per condition. FNG/Lrh-FQ cottage cheese A and B are biological replicates, produced in separate vats with different FNG and Lrh-FQ lot numbers.

During a search for bacterial solutions to replace sorbate or other additives to inhibit growth of *Listeria* spp. in cottage cheese, several *L. rhamnosus* strains were found to severely limit growth of *Listeria* spp. in a model for warm filled cottage cheese when added as an adjunct culture to the FNG-10 cottage cheese starter, consisting of a variety of *Lactococcus lactis* and *Streptococcus thermophilus* strains. To validate the anti-listerial activity of *L. rhamnosus* FreshQ^®^ Cheese + LI 1 strain (from here on referred to as Lrh-FQ) which also showed a desirable low post-acidification profile, its performance was tested in creamed cottage cheese prepared according to US guidelines and contaminated with a mixture of six acid-adapted *L. monocytogenes* strains associated with listeriosis outbreaks and/or isolated from various food products. Two types of cottage cheeses were generated: FNG cottage cheese (control) and FNG/Lrh-FQ cottage cheese. The *L. monocytogenes* mixture was added immediately after mixing curd with cream dressing, after which the cheeses were stored immediately at 7.2 °C according to FDA NCIMS PMO requirements or according to industry practices for warm filled cottage cheese, during which the cheeses are typically cooled from 12.8 to 7.2 °C over a period of 72 h. The increase in CFU counts and pH was followed in each cottage cheese over a period of 60-days (Figure 1). 

The various curds and cream dressings, as well as the composite cottage cheeses did not show any large deviations across both trials in terms of moisture (82.31% ± 0.69%), NaCl percentage (1.11 ± 0.04%, ranging from 1.07–1.88%), water activity (0.988 ± 0.002), or initial pH (5.24 ± 0.10, ranging from 5.08–5.35) (Table 3). 

The initial inoculation level of *L. monocytogenes* for each cheese (3.81 ± 0.25 log CFU/g) was used as a baseline to calculate the increase in CFU counts over time. Independent of the cooling procedure, the FNG cottage cheeses supported a steady increase in *L. monocytogenes* numbers, which reached a 1.6-to-2.6-log increase from Day 0 to Day 7 and further increased significantly up to Day 14 (Figure 1A,B). In FNG/Lrh-FQ cottage cheese, growth of *L. monocytogenes* appeared similar to that in FNG cottage cheeses up to Day 4, reaching a maximum of 1-log CFU/g increase in FNG/Lrh-FQ cottage cheese cooled according to industry practices (Figure 1A,B). However, in all FNG/Lrh-FQ cottage cheeses, the *L. monocytogenes* population started to decline after Day 4 and remained suppressed for the duration of the 60-day study (Figure 1A,B). As the acidification profiles of FNG and FNG/Lrh-FQ cottage cheeses remained comparable for a period up to 30 days (Figure 1C,D), acidification cannot solely account for the observed growth delay in cottage cheese prepared in the presence of Lrh-FQ. Rather, another mode of action evokes an extra margin of protection.

### 3.2. Lrh-FQ Does Not Produce Antilisterial Bacteriocins under Relevant Conditions

**Figure 2:**
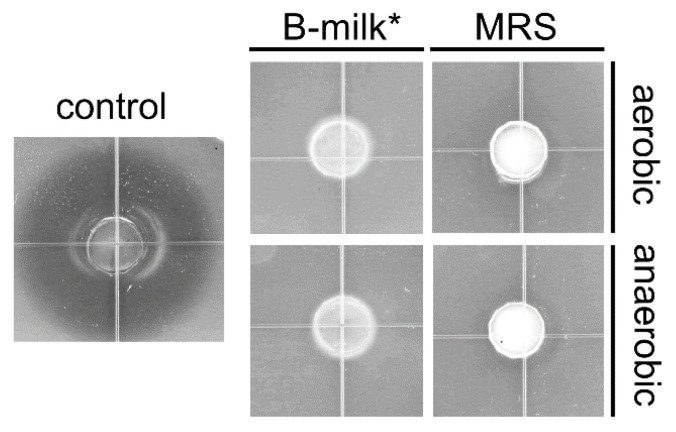
Analysis of anti-listerial compounds secreted by Lrh-FQ using soft-agar well diffusion assays with *L. innocua* BL 86/26 as an indicator strain. Tested were saturated cultures of Lrh-FQ grown in MRS or B-milk supplemented with 0.5% glucose and 0.5% tryptone (B-milk*) under aerobic and anaerobic conditions, as well as a saturated culture of a sakacin P-producing strain grown in MRS (control).

*L. rhamnosus* strains have been reported to produce bacteriocins, of which several have been shown to display anti-listerial activity [44,45,46]. To investigate if Lrh-FQ inhibits *L. innocua*—a species often used as a non-virulent and non-pathogenic surrogate of *L. monocytogenes*—through the production of bacteriocins, several well diffusion assays were performed in which anti-listerial activity of Lrh-FQ cultures grown in rich nutrient broth or milk was tested. Furthermore, whey was collected from milk fermented with the FNG starter culture alone or in combination with Lrh-FQ. None of the tested settings resulted in inhibition zones of *L. innocua* (Figure 2). Therefore, we concluded that Lrh-FQ does not produce bacteriocins active against *L. innocua*, at least not under the tested conditions, and that the observed *Listeria* inhibition is effectuated by another mechanism.

### 3.3. Growth of L. innocua in Cottage Cheese Is Limited by a Combination of Mn Availability and pH

Certain lactobacilli interfere with growth of yeast and mold in yoghurt through scavenging of Mn [37]. To examine if growth of *Listeria* spp. can be hampered by depletion of Mn rather than secretion of a specific compound, we initially analyzed if *L. innocua* grows in whey collected from milk fermented with FNG-10 starter culture alone, FNG-10 in combination with Lrh-FQ, or Lrh-FQ alone. In a series of experiments, we found that: (1) whey obtained from milk fermented in the presence of Lrh-FQ restricted growth of *L. innocua*; (2) this effect could be restored by adding MnCl_2_; and (3) Mn levels as they naturally occur in milk might already limit growth of *Listeria* (Supplementary Materials Figure S1).

The filter-sterilized whey samples no longer contain the original starter culture and/or Lrh-FQ. In addition, access to key nutrients other than Mn, such as amino acids, are more limited in whey compared to curd (Supplementary Materials Figure S1). These samples therefore do not provide information on additional barriers established by competitive exclusion and acidification as it takes place in cottage cheese after mixing the curd and the cream dressing. Thus, to further address how Mn affects growth of *L. innocua,* we performed two different challenges in cottage cheese models prepared from FNG or FNG/Lrh-FQ-based curd. 

**Figure 3:**
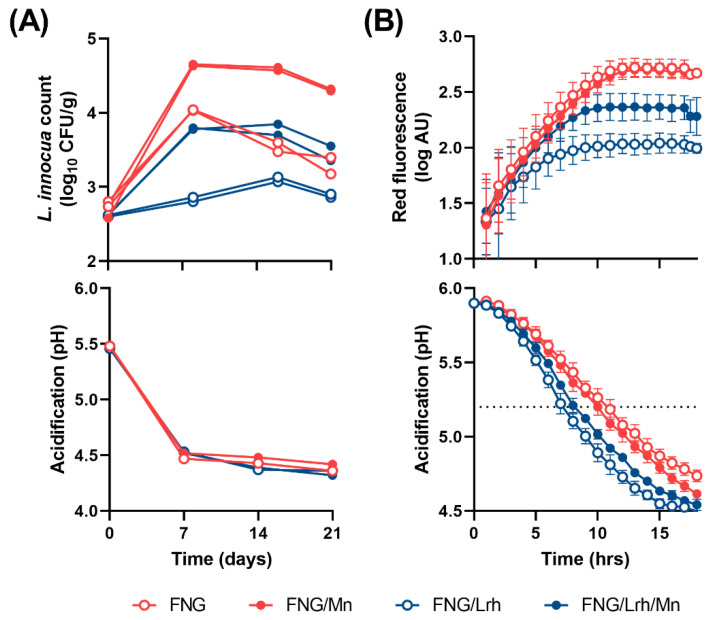
Growth of *L. innocua* and development of pH in cottage cheese models. (**A**) Progression of *L. innocua* counts in CCM prepared from milk fermented with FNG +/− Lrh-FQ +/− 6 mg/L MnCl_2_ (top panel) and the corresponding acidification profile (bottom panel). (**B**) Development of red fluorescence by RFP-tagged *L. innocua* after inoculation in pH-adjusted CBM +/− 6 mg/L MnCl_2_ during incubation at 30 °C (top panel) and acidification profiles of non-contaminated CBM samples under similar conditions (bottom panel). Red fluorescence was normalized using fluorescence values measured at timepoint 0, whereas acidification curves were normalized to start at pH 5.8 after converting the hue of indicator dye to pH.

In the first challenge, *L. innocua* was added to a final concentration of 3-log CFU/g in a cottage cheese model (CCM) during mixing of curd with the cream dressing. The resulting cottage cheeses were stored at 12 °C for the initial 6 days and at 7 °C for the remaining days. The pH values and *L. innocua* CFU counts were monitored for 21 days at 7-day intervals. Although no difference in pH was detected, *L. innocua* was successfully inhibited in the FNG/Lrh-FQ-based CCM compared to the FNG-based CCM (Figure 3A). Moreover, supplementation with 6 mg/L MnCl_2_ prior to milk fermentation supported a >1-log increase in CFU counts in the FNG/Lrh-FQ-based CCM, indicating that Mn levels are limiting the growth of *L. innocua*.

In a second challenge, the FNG or FNG/Lrh-FQ-based curds were mixed with B-milk (CBM) after which the pH was adjusted to 5.8–6. The different CBM samples were then incubated at 30 °C with the addition of 0.005 OD_600_ units (~6.5-log CFU/mL) of *L. innocua* expressing a red fluorescent protein (RFP) to follow growth or a pH indicator dye to follow acidification in real-time. The deviating temperature and pH values were chosen to promote growth of *L. innocua.* Hardly any fluorescence developed in CBM-FNG/Lrh-FQ, but addition of MnCl_2_ to a final concentration of 6 mg/L at the time of mixing resulted in increased fluorescence, indicating stimulation of *L. innocua* growth (Figure 3B). Most fluorescence developed in the CBM-FNG samples, regardless of Mn addition. Acidification was slightly faster in CBM-FNG/Lrh-FQ than in CBM-FNG samples. When aligning acidification with fluorescence profiles (Figure 3B), it becomes clear that RFP expression, and thus vegetative growth, comes to a halt whenever a non-permissive pH below approximately 5.2 is reached. However, up until that pH value is reached, the level of Mn forms a major growth-limiting factor. 

### 3.4. Listeria spp., Unlike LAB Strains Derived from FNG-10, Require Mn for Growth in Chemically Defined Media

**Figure 4:**
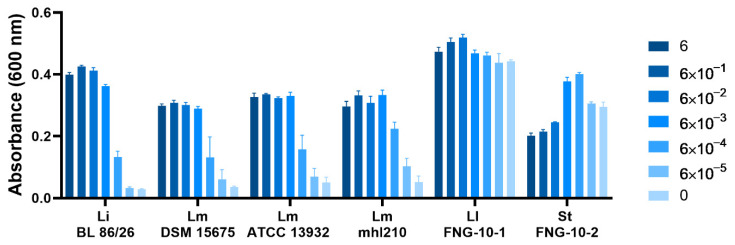
The effect of Mn on growth of *Listeria* spp., *L. lactis* FNG-10-1 or *S. thermophilus* FNG-10-2 in CDM. Depicted are the corrected and averaged OD_600_ values of 20-h-old cultures grown in Mn-free CDM supplemented with the indicated MnCl_2_ concentration (in mg/L). All cultures were grown in six-fold as standing 200-µL cultures at 30 °C in 96-well plates covered with a transparent seal. Li = *L. innocua*, Lm = *L. monocytogenes*, Ll = *L. lactis*, St = *S. thermophilus*. Growth curves and doubling times are provided in Supplementary Materials Figure S2.

Since the availability of Mn in the extracellular environment appears to be indispensable for growth of *Listeria* spp., we examined to what extent *L. innocua* cells and, more importantly, *L. monocytogenes* cells, rely on Mn for optimal growth. To do so, the growth of *L. innocua* and three *L. monocytogenes* strains was followed in CDM containing various Mn concentrations. After 20 h of cultivation at 30 °C, a clear relation between reduced growth and low Mn concentrations was observed (Figure 4). Growth of *L. innocua* and *L. monocytogenes* became hampered when the added MnCl_2_ concentration was reduced to 6 × 10^−3^ mg/L. Importantly, growth was even more impeded at concentrations below 6 × 10^−4^ mg/L. Since the individual components used to prepare the CDM might be contaminated with traces of Mn, we expect the absolute requirement for Mn to be higher than observed here. When examining the growth curves and doubling times of the *Listeria* strains, an initial increase in absorbance can be observed independent of the Mn concentration (Supplementary Materials Figure S2). This is presumably caused by carry over of residual intracellular Mn accumulated during the pre-culturing step in rich nutrient broth. However, once the strains had adapted to the medium, growth rates ceased or increased under limiting or sufficient Mn concentrations, respectively (Supplementary Materials Figure S2). 

To establish if Mn depletion also interferes with the FNG starter culture, growth of two strains that are part of the FNG-10 culture, *L. lactis* FNG-10-1 and *S. thermophilus* FNG-10-2, was monitored under comparable conditions (Figure 4 and Supplementary Materials Figure S2). Unlike the *Listeria* strains, growth of *L. lactis* and *S. thermophilus* was not inhibited by Mn limitation. This indicates that extracellular depletion of Mn by Lrh-FQ results in *Listeria*-specific growth hurdles and does not affect a typical cottage cheese starter culture. Rather, *S. thermophilus* FNG-10-2 seems to become hampered when MnCl_2_ levels exceed 6 × 10^−3^ mg/L.

### 3.5. Addition of Mn Restores Growth of Listeria spp. in Industrial Cottage Cheese

As a validation that Mn depletion by Lrh-FQ also reduces outgrowth of *L. monocytogenes* in industrial cottage cheeses, we prepared FNG or FNG/Lrh-FQ warm filled cottage cheeses using the FNG-20 starter culture according to the US guideline (cottage cheese II in methods section) and evaluated the effect of 6 mg/L MnCl_2_ addition on the growth of a cocktail of three milk-adapted *L. monocytogenes* (3.31 ± 0.24 log CFU/g). As evidenced above, the low Mn content present in milk already sets limits to the growth of *Listeria* and, furthermore, induces the upregulation of Mn importers in *Listeria* cells [47]. We therefore speculated that the *L*. *monocytogenes* strain-mixture pre-grown in milk would show improved competition for Mn and would thus be less inhibited by Lrh-FQ.

The composite cottage cheeses did not show any large deviations in terms of moisture (85.53 ± 0.36%), NaCl percentage (1.04 ± 0.01%), or initial pH (5.31 ± 0.07, ranging from 5.37–5.24) (Table 3). 

**Figure 5:**
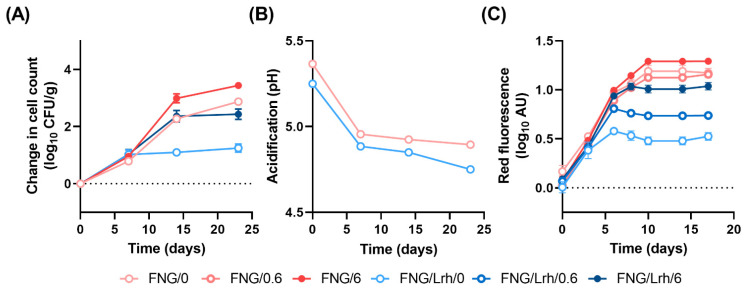
Effect of Mn supplementation on growth of *Listeria* spp. in cottage cheese prepared with or without Lrh-FQ. Depicted in (**A**) are changes in *L. monocytogenes* CFU/g in FNG or FNG/Lrh-FQ (FNG/Lbh) industrial cottage cheeses to which no or 6 mg/L MnCl_2_ was added after mixing. The corresponding acidification profiles of the non-contaminated control cheeses are shown in (**B**). Averaged measurements were obtained from three cheeses per condition. (**C**) The development of red fluorescence by RFP-tagged *L. innocua* after inoculating industrial cottage cheeses supplemented with no, 0.6 mg/L or 6 mg/L MnCl_2_, and incubated at 10 °C for 17 days.

The FNG and FNG/Lrh-FQ cottage cheeses were stored at 7 °C over a period of 3 weeks during which pH values and *L*. *monocytogenes* counts were monitored on a weekly basis (Figure 5A,B). All cottage cheese formulations supported an increase of *L. monocytogenes* of around 1-log CFU/mL at Day 7. In agreement with previous findings, the *L. monocytogenes* population remained unaltered after Day 7 in FNG/Lrh-FQ cottage cheese without added Mn, but had increased further at Day 14 in all other cheeses (Figure 5A). This shows that Lrh-FQ also reduces outgrowth of milk-adapted *Listeria* in cottage cheese through competitive exclusion of Mn.

Finally, we employed the RFP-tagged *L. innocua* strain to study its growth in the same FNG and FNG/Lrh-FQ cottage cheeses, stored at 10 °C for 17 days, as a function of supplementation with 0, 0.6 or 6 mg/L MnCl_2_ (Figure 5C). The observed surge in fluorescence in FNG cottage cheese over time indicates that substantial growth of *L. innocua* took place. The presence of Lrh-FQ on top of FNG in non-supplemented cottage cheese did not result in an increase in fluorescence beyond the first week, further displaying the strong additional effect of Mn depletion next to acidification in inhibiting growth of *Listeria*. Supplementation with 6 mg/L MnCl_2_ to FNG/Lrh-FQ cheese resulted in an almost full recovery of *L. innocua* growth compared to that of FNG cottage cheese. Unlike what we observed in CDM, the supplementation of FNG/Lrh-FQ cottage cheese with 0.6 mg/L MnCl_2_ was not sufficient to restore fluorescence to control levels. This indicates that in the actual application, Mn becomes limited faster than in the CDM model, either through ongoing Mn-scavenging by Lrh-FQ, interactions of Mn with milk components, or a combination thereof.

## 4. Discussion

The combined results described in this study show that the addition of *L. rhamnosus* Lrh-FQ to milk fermentations limits the outgrowth of *L. monocytogenes* in cottage cheese during prolonged storage at refrigerated temperatures. The mechanism behind the anti-listerial activity was found to rely on competitive exclusion realized by depletion of available Mn by Lrh-FQ. Without the addition of Lrh-FQ, *Listeria* spp. were able to grow substantially in cottage cheese until a non-permissive pH was reached. Our study indicates that depletion of bioavailable Mn in cottage cheese establishes an additional protection until that pH is reached. 

Currently available bacterial solutions for the inhibition *L. monocytogenes* in cottage cheese mainly consist of bacterio-cinogenic strains, such as *L. lactis* starters that produce bacteriocins such as nisin A, nisin Z, lacticin 481 or lacticin 3147 [48,49]. Alternatively, semi-purified bacteriocins have been employed, like nisin, enterocin, lacticin 481 or class IIa pediocin-like bacteriocins [50,51,52,53,54,55,56]. Besides bacteriocins, some secondary metabolites, including reuterin produced by *Lactobacillus reuteri*, resulted in growth repression of *L. monocytogenes* in dairy products including cottage cheese [57,58]. Others, such as diacetyl, were reported to be less effective against *Listeria* [58]. Although very effective, resistant mutants of *Listeria* against bacteriocins and other compounds can arise [59,60,61]. Inhibitory modes of action through competitive exclusion, on the other hand, do not readily lead to such resistance, which therefore might constitute a more sustainable solution. We confirmed that Lrh-FQ does not secrete potent anti-listerial secondary metabolites or bacteriocins when grown in MRS or milk. To our knowledge, this is the first anti-listerial mode of action in a food product effectuated by a LAB strain independent of bacteriocin and/or secondary metabolite production but through competitive exclusion of Mn. 

Recently, we elucidated that specific *L. paracasei* and *L. rhamnosus* species can protect yoghurt from yeast and mold growth through the expression of MntH1, a Mn importer that enables these cells to carry out hyperactive Mn scavenging [37]. Deletion of the *mntH1* in *L. paracasei* led to the abolishment of its bioprotective effect. Thus, through the action of this transporter, hyperactive Mn-scavenging lactobacilli are believed to quickly accumulate and subsequently store high Mn levels, while rapidly depleting the extracellular milieu of this trace metal. For instance, Mn levels in milk-to-yoghurt fermentations in the presence of a hyperactive Mn-scavenging *L. paracasei* strain were found to drop from 2–3 × 10^−2^ mg/L to below the method’s detection limit of 3 × 10^−3^ mg/L [37,62,63]. In the CDM trials performed in the current study, we found that Mn becomes limiting for *Listeria* spp. at a MnCl_2_ concentration between 6 × 10^−3^ and 6 × 10^−4^ mg/L. Considering that trace amounts of Mn might still be present in CDM through contaminations of chemicals, these values correspond to the lower detection limit reported for yoghurt. We thus expect a similar drop in Mn availability to take place when employing Lrh-FQ in the manufacture of cottage cheese as found in yoghurt, even though during cottage cheese manufacture an influx of new nutrients, including Mn, occurs when the cream is mixed with the already nutrient-depleted curd. 

For most experiments presented in this study, we have utilized *Listeria* strains pre-grown in rich-nutrient broth. However, in industrial food plants, contaminations with *Listeria* often emerge from polluted surfaces or from raw milk [16,21]. Under the former situation, listerial cells are already depleted of nutrients and in a physiologically distinct state than the vegetative cells used here [64]. We therefore expect such cells to have an even greater disadvantage when inoculated in cottage cheese. It would thus be of interest to examine if a difference in *Listeria* outgrowth occurs when different states of inoculum material are used. 

With respect to survival in milk, gene expression profile studies of *L. monocytogenes* strain F2356 revealed that genes encoding the two main Mn transporters, MntH (*lmo1443*) and MntABC (*lmo1875-1877*), were among the highest upregulated genes when grown in ultrahigh-temperature-processed (UHT) skimmed milk compared to BHI broth [47]. These transporters are generally upregulated in bacteria under Mn-limiting and/or oxidative stress conditions; Mn is required as a co-factor for many bacterial enzymes involved in the detoxification of reactive oxygen species (reviewed by [65,66]). At least one superoxide dismutase of *L. monocytogenes* requires Mn as a cofactor [67]. However, given that the upregulation of the Mn transporters did not alter the sensitivity towards hydrogen peroxide when the strain was grown in BHI or UHT skimmed milk, the upregulation of Mn transport genes is likely to be an effect of the limiting concentration of Mn in milk [47]. This data is in line with our findings that supplementation of Mn to milk prior to fermentation with the starter culture alone improves growth of *L. innocua*. Based on this data, we also speculate that *Listeria* pre-grown in milk is better adapted to compete for Mn through upregulated Mn import and therefore more difficult to inhibit through Mn depletion. Preliminary results indicate that pre-growth in rich nutrient broth instead of milk indeed results in less growth of *Listeria* in FNG/Lrh-FQ cottage cheese. Nevertheless, even when *Listeria* spp. are pre-adapted to milk and low Mn levels, the presence of Lrh-FQ poses a substantial hurdle to the growth of *Listeria*. 

Another question that arises regarding the rather Mn-independent *L. lactis* and *S. thermophilus* starter culture strains is why *Listeria* is particularly sensitive towards Mn depletion. In pathogen-host interactions, transition metals are often kept at low concentrations at infection sites and inside neutrophils through the action of several metal chelating factors secreted by the host. One of these is the protein calprotectin, which exerts antimicrobial activity through the action of Mn, iron or zinc scavenging [68,69,70,71]. Calprotectin is induced during enteric infections with *Listeria* in rodents [72], and inhibits growth of *L. monocytogenes* [73,74]. This was initially addressed to abrogation of zinc availability, but, based on the results described here, might very well be related to Mn depletion [73,75].

When evaluating the Mn requirement of other Gram-positive pathogens for growth, two distinct patterns can be observed. On the one hand, there are species such as *Enterococcus faecalis* and *Streptococcus mutans* that do not depend on the presence of Mn for growth in CDM [76,77]. Our results support the notion that this is also true for related species such as the *L. lactis* and *S. thermophilus* strains found in starter cultures. However, growth becomes hampered in *E. faecalis* and *S. mutans* mutants that lack high-affinity Mn uptake mechanisms in Mn-restricted environments [76,77]. This would also be interesting to examine for the starter culture species. On the other hand, bacilli and staphylococci, genera more closely related to the *Listeria* genus, rely on low levels of extracellular Mn for growth in CDM [78,79], and are more sensitive to Mn restriction. It might well be that *Listeria* spp. require Mn for glycolytic enzymes for glucose utilization, as is observed for several other Gram-positive bacteria including *S. aureus* and *B. subtilis*. These contain an isoform of phosphoglycerate mutase, among other glycolytic enzymes, that is more active or can only function with Mn as co-factor [78,80,81]. 

## 5. Conclusions

A substantial inhibition of *Listeria* growth in dairy products—that inherently have low Mn concentrations—can be achieved through the addition of Lrh-FQ, a *L. rhamnosus* strain that depletes available Mn to levels too low to support growth of *L. monocytogenes*. This mode of action differs substantially from previously reported bacteriocin-based solutions. The depletion of Mn effectively protects cottage cheese from outgrowth of contaminations after creaming, where a relatively short time frame exists in which the pH, nutrient availability and temperature are permissible for growth of *Listeria* spp. Moreover, it is plausible that hyperactive Mn-scavenging LAB such as Lrh-FQ inhibit *Listeria* and potentially more pathogenic and/or spoiler bacteria in other dairy and Mn-poor food products.

## Figures and Tables

**Table 1:** Strains used in this study. Serovars and origins are given for *L. monocytogenes* strains.

Species	Strain	Culture Collection	Serovar #	Origin
*Listeria monocytogenes*	mhl210	IVH KU	ND	Isolated from pork
*Listeria monocytogenes*	ATCC 13932	ATCC	4b	Clinical isolate of spinal fluid of child with meningitis
*Listeria monocytogenes*	DSM 15675	DSMZ	4b	Isolated from soft cheese
*Listeria monocytogenes*	FSL R2-501	ILSI Cornell	4b	Hispanic-style cheese isolate, associated with NC outbreak
*Listeria monocytogenes*	FSL R2-500	ILSI Cornell	4b	Clinical isolate, associated with NC Hispanic-style cheese outbreak
*Listeria monocytogenes*	LM101	UW-Madison FRI	4b	Hard salami isolate
*Listeria monocytogenes*	LM310	UW-Madison FRI	4b	Goat cheese isolate associated with illness
*Listeria monocytogenes*	LM301	UW-Madison FRI	1/2a	Heat-treated Cheddar cheese isolate
*Listeria monocytogenes*	LM108	UW-Madison FRI	1/2b	Hard salami isolate
*Listeria innocua*	BL 86/26	TNO		
*Listeria innocua*	BL 86/26 + pEFB001	This study		
*Laticaseibacillus rhamnosus*	FreshQ^®^ Cheese +LI 1	Chr. Hansen A/S		
*Lactococcus lactis*	MG1363	MoBiTec GmbH		
*Lactococcus lactis*	MG1363 + pEFB001	This study		
*Lactococcus lactis*	FNG-10-1	Chr. Hansen A/S		
*Streptococcus thermophilus*	FNG-10-2	Chr. Hansen A/S		

# ND = not determined. *L. monocytogenes* serovars 1/2a, 1/2b and 4b are associated with listeriosis.

**Table 2:** Oligonucleotides used in this study.

Name	Sequence (5′ → 3′)
EFB0057	GAAGAAGGTTTTTATATTACAGCTCCAGATCTAGCGCTATAGTTGTTGACAG
EFB0060	CTTGGTTTTCTAATTTTGGTTCAAAGAAAGCTTTTATTTGTACAGCTCATCC
EFB0061	GGATGAGCTGTACAAATAAAAGCTTTCTTTGAACCAAAATTAGAAAACCAAG
EFB0062	CTGTCAACAACTATAGCGCTAGATCTGGAGCTGTAATATAAAAACCTTCTTC
EFB0055	CAACATCTTCGCTGCAAAGC
EFB0056	CTCTATTCAGGAATTGTCAG

**Table 3:** Proximate analysis of cottage cheeses prepared and used in this study.

**Cottage Cheese I (Using FNG-10)**				
**Formulation**	**% Moisture**	**Water Activity**	**pH**	**% NaCl**
FNG curd	81.38 ± 0.15	0.998 ± 0.002	4.49 ± 0.00	0.06 ± 0.01
FNG/Lrh-FQ curd	80.19 ± 0.14	0.998 ± 0.002	4.52 ± 0.01	0.05 ± 0.00
Dressing	82.84 ± 0.02	0.983 ± 0.000	6.64 ± 0.01	2.16 ± 0.03
FNG cottage cheese composite	83.19 ± 0.13	0.987 ± 0.001	5.09 ± 0.01	1.08 ± 0.08
FNG/Lrh-FQ cottage cheese composite	82.61 ± 0.16	0.991 ± 0.003	5.14 ± 0.02	1.10 ± 0.03
**Cottage Cheese II (Using FNG-20)**				
**Formulation**	**% Moisture**	**Water Activity**	**pH**	**% NaCl**
FNG cottage cheese composite	85.33 ± 0.15	ND	5.37 ± 0.05	1.04 ± 0.01
FNG/Lrh-FQ cottage cheese composite	85.23 ± 0.11	ND	5.26 ± 0.05	1.04 ± 0.00

ND = not determined.

## Data Availability

Data is contained within the article or Supplementary Materials.

## Supplementary Materials

The following are available online at https://www.mdpi.com/article/10.3390/foods10061353/s1, Table S1: Composition of Mn-deficient CDM-a and CDM-b, Figure S1: Culture density of *L. innocua* grown for 20 h in mixtures containing filter-sterilized whey of milk fermented with FNG, DSM33386 or both, Figure S2: The effect of Mn on the growth rate of various individual *Listeria* strains and two individual strains obtained from the FNG starter culture.
