# Supplementary material for: Lacticaseibacillus rhamnosus Impedes Growth of Listeria spp. in Cottage Cheese through Manganese Limitation
Source: Foods. 2021 Jun 11;10(6):1353. doi: 10.3390/foods10061353 (PMC8230772; doi:10.3390/foods10061353)
Supplement: Supplementary file 1 [file foods-10-01353-s001.zip › foods-1227388-supplementary.pdf]

Supplementary Files to

Lacticaseibacillus rhamnosus impedes growth of *Listeria* spp. in cottage cheese through manganese limitation

Lieke A. van Gijtenbeek[1], Quinn Singer[2], Louise E. Steffensen[1], Shannon Neuens[1], Helle S. Guldager[1], Susanne Bidstrup[1], Tina Høgholm[1], Mikkel G. Madsen[1], Kathleen Glass[2], Solvej Siedler*[1]

[1] Chr. Hansen A/S, Bøge Alle 10-12, Hørsholm DK-2970, Denmark
[2] Food Research Institute, 1550 Linden Drive, University of Wisconsin-Madison, Madison, WI, 53706, USA
* Correspondence: dksosi@chr-hansen.com

Table S1. Composition of manganese-deficient CDM-a and CDM-b. All components were dissolved in MilliQ water as various stock solutions and mixed, after which the pH was set to 6.8 with KOH and the volume was adjusted to 1 L and filter-sterilized.

Component	CDM-a	CDM-b
**Basal components**		
**Glucose**	27.76 mM	27.76 mM
**Sodium acetate**	15.24 mM	12.19 mM
**Ammonium citrate**	2.47 mM	2.47 mM
**K_2HPO_4**	5.74 mM	14.35 mM
**KH_2PO_4**	36.74 mM	22.04 mM
**$NaHCO_3$**	-	4.83 mM
**Urea**	-	2 mM
**Amino acids**		
**L-Alanine**	3.4 mM	0.90 mM
**L-Arginine**	1.1 mM	0.46 mM
**L-Asparagine**	0.8 mM	0.61 mM
**L-Aspartate**	0.8 mM	0.60 mM
**L-Glutamate**	2.1 mM	0.55 mM
**L-Glutamine**	0.7 mM	0.55 mM
**Glycine**	2.7 mM	1 .07 mM
**L-Histidine**	0.3 mM	0.52 mM
**L-Isoleucine**	0.8 mM	0.61 mM
**L-Leucine**	0.8 mM	0.61 mM
**L-Lysine**	1.4 mM	0.55 mM
**L-Methionine**	0.7 mM	0.54 mM
**L-Phenylalanine**	1.2 mM	0.48 mM
**L-Proline**	2.6 mM	0.70 mM
**L-Serine**	2.8 mM	0.76 mM
**L-Threonine**	1.7 mM	0.67 mM
**L-Tryptophan**	0.5 mM	0.39 mM
**L-Tyrosine**	0.3 mM	0.44 mM
**L-Valine**	0.9 mM	0.68 mM
**Cysteine**	0.8 mM	4.13 mM

Component	CDM-a	CDM-b
**Vitamins**		
**Biotin**	0.41 µM	0.82 µM
**Folic acid**	2.27 µM	0.45 µM
**Pyridoxal·HCl**	9.82 µM	4.91 µM
**Riboflavin**	2.66 µM	1.33 µM
**Thiamine·HCl**	2.96 µM	1.48 µM
**Nicotinamide**	8.19 µM	4.46 µM
**Cyanocobalamin**	-	0.37 µM
**p-Aminobenzoic acid**	-	3.65 µM
**Ca-pantothenate**	2.10 µM	22.67 µM
**Lipoic acid**	9.69 µM	2.42 µM
**L-ascorbic acid**	2.84 mM	-
**Biotin**	0.41 µM	0.82 µM
**Folic acid**	2.27 µM	0.45 µM
**Pyridoxal·HCl**	9.82 µM	4.91 µM
**Riboflavin**	2.66 µM	1.33 µM
**Thiamine·HCl**	2.96 µM	1.48 µM
**Nicotinamide**	8.19 µM	4.46 µM
**Bases**		
**Adenine**	-	74.00 µM
**Guanine**	-	66.17 µM
**Uracil**	-	89.21 µM
**Xanthine**	-	65.74 µM
**Metals**		
**$MgCl_2 \cdot 6H_2O$**	0.98 mM	0.98 mM
**$CaCl_2 \cdot 2H_2O$**	0.34 mM	0.34 mM
**$ZnSO_4 \cdot 7H_2O$**	17.39 µM	17.39 µM
**$CoSO_4 \cdot 7H_2O$**	8.89 µM	8.89 µM
**$CuSO_4 \cdot 5H_2O$**	10.01 µM	10.01 µM
**$(NH_4)_6Mo_7O_{24} \cdot 4H_2O$**	2.02 µM	2.02 µM
**$FeCl_3 \cdot 6H_2O$**	5.55 µM	5.55 µM
**$FeCl_2 \cdot 4H_2O$**	12.58 µM	12.58 µM

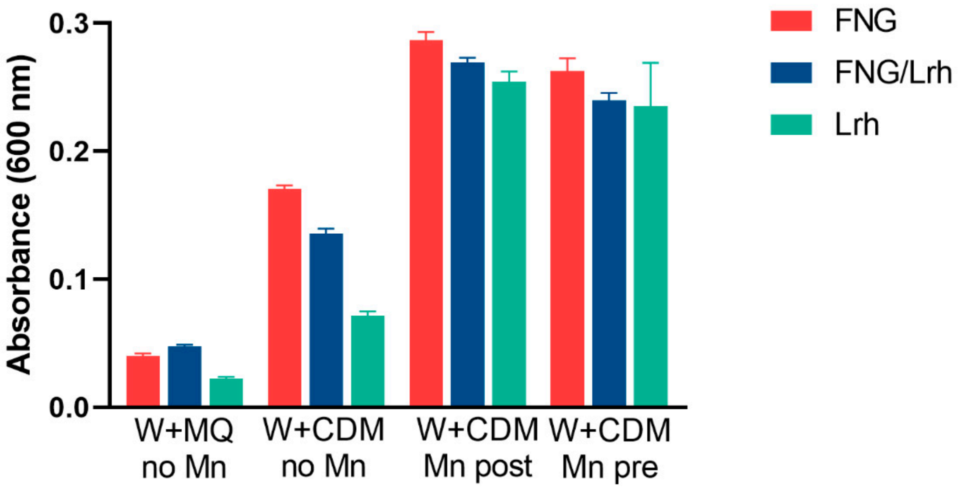

Figure S1. Culture density of *L. innocua* grown for 20 hr in mixtures containing filter-sterilized whey of milk fermented with FNG, Lrh-FQ or both. *L. innocua* BL 86-26 was grown in a pH-adjusted whey/MilliQ mixture (W+MQ, no Mn), a whey/CDM-a mixture (W+CDM, no Mn), a whey/CDM-a mixture supplemented with 6 mg/L $MnCl_2$ (W+CDM, Mn post) or a whey/CDM-a mixture using whey obtained for milk to which 6 mg/L $MnCl_2$ was added prior to fermentation (W+CDM, Mn pre). The whey was mixed in a 1-to-1 ratio with MilliQ or CDM, adjusted to a—for *L. innocua*—growth permissible pH of 6.0, filter sterilized, provided with 0.5% glucose, and inoculated with *L. innocua.* Incubation was carried out in a 96-well plate for 20 hr at 30°C. Shown are averaged absorbance values of pentaplicate measurements corrected using background values of blank medium controls.

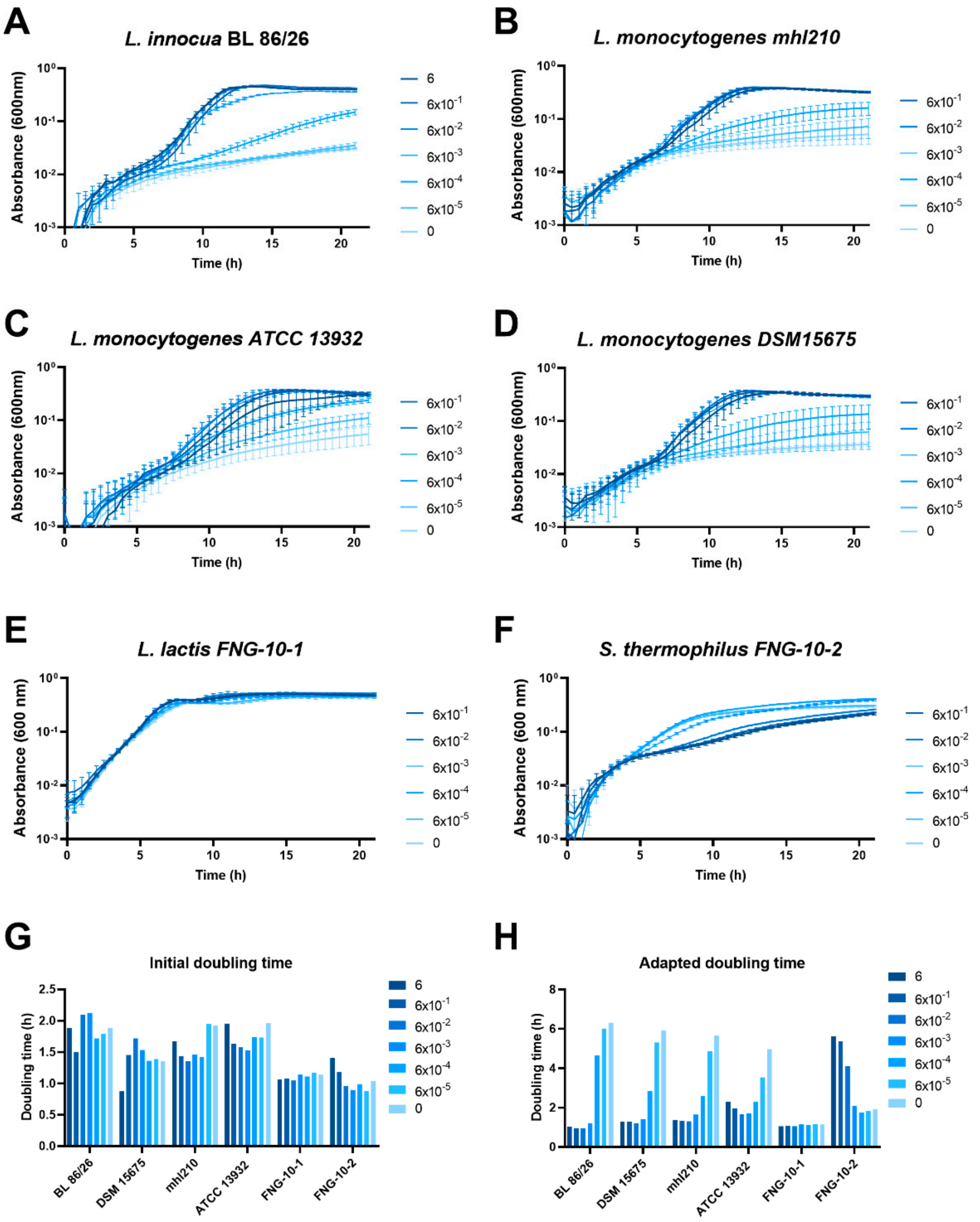

Figure S2. The effect of Mn on the growth rate of various *Listeria* strains and two individual strains obtained from the FNG-10 starter culture. (A-F) Growth curves of Li = *L. innocua*, Lm = *L. monocytogenes*, Ll = *L. lactis*, St = *S. thermophilus* in in Mn-free CDM supplemented with indicated $MnCl_2$ concentration (in mg/L). Depicted are corrected and averaged OD_{600} values. All cultures were grown as standing 200-µL cultures 30°C in six-fold in 96-well plates covered with a transparent seal. Li, Lm, and Ll were grown in CDM-a, whereas St was grown in CDM-b. **(G)** Doubling times calculated over the initial period of growth during which cells pre-grown in in rich nutrient broth are adapting to CDM from pre-growth (derived from the 0–to–7 hr timepoints for *Listeria* strains, and from 0–to–3 hr timepoints for FNG strains). (**H**) Doubling times calculated after adaptation to manganese-depleted CDM (derived from >7 hr or >3 hr timepoints during exponential growth phase for *Listeria* strains or FNG strains, respectively).
